# HCV Infection and B-Cell Lymphomagenesis

**DOI:** 10.1155/2011/835314

**Published:** 2011-07-20

**Authors:** Masahiko Ito, Hideki Kusunoki, Keiko Mochida, Kazunari Yamaguchi, Toshiaki Mizuochi

**Affiliations:** ^1^Department of Research on Blood and Biological Products, National Institute of Infectious Diseases, 4-7-1 Gakuen, MusashiMurayama-shi, Tokyo 208-0011, Japan; ^2^Department of Infectious Diseases, Hamamatsu University School of Medicine, 1-20-1 Handayama, Higashi-ku, Hamamatsu 431-3192, Japan; ^3^Department of Bacterial Pathogenesis and Infection, National Institute of Infectious Diseases, 4-7-1 Gakuen, MusashiMurayama-shi, Tokyo 208-0011, Japan

## Abstract

Hepatitis C virus (HCV) has been recognized as a major cause of chronic liver diseases worldwide. It has been suggested that HCV infects not only hepatocytes but also mononuclear lymphocytes including B cells that express the CD81 molecule, a putative HCV receptor. HCV infection of B cells is the likely cause of B-cell dysregulation disorders such as mixed cryoglobulinemia, rheumatoid factor production, and B-cell lymphoproliferative disorders that may evolve into non-Hodgkin's lymphoma (NHL). Epidemiological data indicate an association between HCV chronic infection and the occurrence of B-cell NHL, suggesting that chronic HCV infection is associated at least in part with B-cell lymphomagenesis. In this paper, we aim to provide an overview of recent literature, including our own, to elucidate a possible role of HCV chronic infection in B-cell lymphomagenesis.

## 1. Introduction

 Hepatitis C virus (HCV) is an enveloped positive-strand RNA virus that belongs to the *Flaviviridae *family [1]. HCV infection is a worldwide problem affecting nearly 200 million people [2] and causes prolonged and persistent diseases in virus carriers, often leading to chronic hepatitis, cirrhosis, and hepatocellular carcinoma [3]. Although the liver is considered to be the primary target of HCV infection, extrahepatic manifestations, such as mixed cryoglobulinemia, which is a systemic immune complex-mediated disorder characterized by B-cell proliferation that may evolve into overt B-cell non-Hodgkin's lymphoma (B-NHL), are often recognized among patients persistently infected with HCV [4, 5]. In fact, epidemiological evidence strongly suggests a close link between chronic HCV infection and B-NHL [6, 7]. The pathogenic role of HCV in B-cell disorders has been suggested in reports wherein a clinical resolution of the B-cell dysfunctions, stated above, was observed after successful anti-HCV treatment using interferon (IFN) [8–10]. Based on such evidences, a possible role of B cells in HCV pathogenesis has been postulated but not yet conclusively demonstrated. 

The objective of this paper is to summarize recent literature focused on the possible involvement of HCV infection in B-cell lymphomagenesis, which could offer new insights into the role of B cells in the pathogenesis of HCV infection.

## 2. Does HCV Infect and Replicate in Peripheral B Cells of Chronic Hepatitis C (CHC) Patients?

 HCV, as the name indicates, has been regarded as a hepatotropic virus. However, the possibility that HCV infects cells other than hepatocytes cannot be excluded. In the early 1990s, the existence of HCV RNA was demonstrated by PCR in not only serum/plasma [11] and liver tissues [12] but also in peripheral blood mononuclear cells (PBMCs) of patients infected with HCV [13, 14]. Muller et al. first reported in 1993 that HCV RNA could be found in B cells [15]. They predicted that PBMC, particularly B cells, could be sites for HCV replication and may serve as reservoirs of HCV infection. Moldvay et al. demonstrated that negative-strand HCV RNA, a replicative intermediate of HCV, was observed in PBMC of patients with CHC (6 of 11) by *in situ* hybridization [16]. Muratori et al. reported negative-strand HCV RNA within PBMC detected by fluorescein-tagged *in situ* RT-PCR (12 of 14 patients with CHC) [17]. Further evidence suggested that HCV replicates in B cells. For example, Morsia et al. demonstrated the replication of HCV in CD19^+^ B cells by detecting the negative-strand RNA although their sample size was very small (1 of 3 patients with CHC was positive) [18]. Around the same time, Pileri et al. demonstrated that the HCV envelope protein E2 binds the CD81 molecule that is expressed on not only hepatocytes but also various cell types including B cells [19]. This finding thus provided a rationale for the notion that HCV infects and replicates in B cells. Several years later, Gong et al. confirmed the existence of negative-strand HCV RNA in PBMC of patients with CHC (14 of 35) [20]. Some argued that the negative-strand HCV RNA in PBMC may be due to mere contamination or passive absorption by circulating HCV in peripheral blood. They successfully excluded this possibility by demonstrating the expression of HCV-encoding protein, NS5, which indicates that HCV not only replicates but also produces HCV protein in PBMC. Their results are in agreement with an earlier study by Sansonno et al. in which HCV core and NS3 proteins were detected in PMBC of patients with CHC [21].

 Occult HCV infection is characterized by the presence of HCV RNA in the liver and the absence of both HCV RNA and anti-HCV antibodies in serum. Castillo et al. detected HCV RNA in PBMC of 40 of 57 (70%) patients with occult HCV infection [22]. In a subsequent report, they confirmed the replication of HCV in PBMC of patients with occult HCV infection by detecting both positive and negative strands of HCV RNA using a strand-specific RT-PCR and *in situ* hybridization techniques [23]. Meanwhile, Januszkiewicz-Lewandowska et al. demonstrated the presence of HCV RNA in PBMC of patients who underwent antiviral chemotherapy and therefore were HCV-serum negative [24]. Collectively, these findings not only favor the notion that PBMC, particularly B cells (discussed later), infected with HCV can serve as reservoirs for persistent HCV infection but are also an alert that PBMC of patients with CHC, including patients with occult CHC, could be potentially infectious even when HCV RNA is negative in their sera. There has been a debate over which cell population in PBMC is the main target for HCV infection. An array of evidence suggests that HCV replicates in various cell types of PBMC, including peripheral dendritic cells, monocytes, and macrophages [25–27]. A recent study by Kondo et al. demonstrated that lymphotropic HCV (SB strain) could infect not only established T-cell lines and B-cell lines but also primary naïve CD4^+^ T cells, suggesting that HCV replication in such T cells suppressed their proliferation and development in Th1 commitment [28]. Under these circumstances, a number of reports have indicated that HCV infects CD81-positive lymphocytes, preferentially B cells [18, 29–31]. Our recent study also clearly demonstrated that HCV RNA and HCV core and NS3 proteins are detected in CD19^+^ but not in CD19^−^ PBMC [32]. Furthermore, Inokuchi et al. confirmed that negative-strand HCV RNA, regarded as a marker of viral replication, was detected in B cells of patients with CHC [33]. Considering this evidence, it can be concluded that HCV infects and replicates in PBMC, particularly in the CD19^+^ B-cell subset, of patients with CHC. An intriguing question has emerged as to whether different HCV variants or B-tropic HCV cause HCV infection in the CD19^+^ B cells of patients with CHC or not. When cDNA sequences derived from RNA isolated from plasma and CD19^+^ B cells of randomly selected patients with CHC were compared, limited variations were found in the internal ribosome entry site (IRES) region (our unpublished data). However, as predicted by a computer program named mfold, these nucleotide substitutions did not affect RNA secondary structure or thermodynamic stability of IRES region [34]. Furthermore, the amino acid sequences in the hypervariable region 1 (HVR1), which directly reflect clonal variations of HCV, did not show any distinct differences between plasma and CD19^+^ B cells of patients with CHC. These results indicate that HCV RNA isolated from CHC B cells is indistinguishable from RNA isolated from plasma of the same patient with CHC (our unpublished data). Sequence polymorphisms located at IRES and HVR1 of E2 were observed in lymphoid cells of individuals with persistent HCV infection, strongly favoring the concept of HCV lymphotropism. Recently, HCV variants observed in B cells showed poor translational activity in hepatocytes but not in B-cell lines, indicating that adaptive mutations had occurred in B cells [35]. However, our results do not support the concept of lymphotropism or B-tropism of HCV in patients with CHC [30] but instead are in good agreement with studies by Muller et al. in which the PCR products obtained from serum and PBMC specimens of an HCV-positive individual were found to have nearly identical sequences [15]. Although the number of clones analyzed was limited, our conclusion that HCV RNA isolated from CD19^+^ B cells is indistinguishable from RNA isolated from the plasma of the same patient with CHC is inconsistent with the concept of B-tropic HCV RNA. Further investigation involving a large number of HCV patients would be necessary to support this conclusion. 

 Overall, the data accumulated to date strongly suggest that HCV infects and replicates in the peripheral B cells of patients with CHC. However, currently it is not known whether a novel HCV strain, B-tropic HCV RNA, preferentially infects peripheral B cells or not. The role of B cells in the pathogenesis of HCV infection is examined in the next section.

## 3. Peripheral B Cells May Serve as Reservoirs for Persistent Infection of HCV

 As described in the previous section, evidence indicates that peripheral B cells in patients with CHC were infected with HCV and thus may serve as HCV reservoirs. This evidence posed a logical question as to how HCV evades the innate antiviral immune responses in B cells. However, this important issue has so far not been formally investigated.

**Figure 1:**
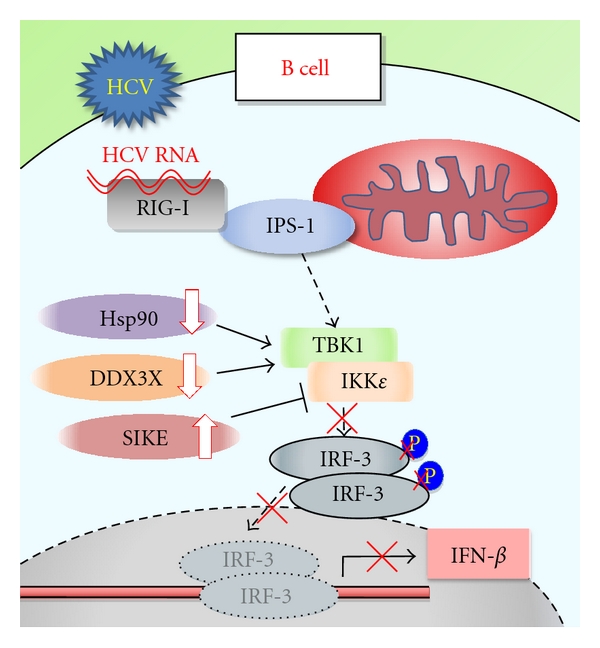
Impaired innate antiviral immunity in B cells of patients with chronic hepatitis C.

Sensing mechanisms for invading viruses in host immune cells consist of toll-like-receptor (TLR-) mediated [36] as well as retinoic-acid-inducible-gene-I-(RIG-I-) mediated [37] pathways. Both pathways culminate in the translocation of IFN regulatory factor-3 (IRF-3) to the nucleus to transcribe the IFN-*β* gene. Type-I IFN, for example, IFN-*β*, plays a critical role in the innate antiviral immune response [38, 39]. In our recent study, it was found that the expression levels of RIG-I and its adaptor molecule, IFN promoter-stimulator 1 (IPS-1), were substantially enhanced in CHC B cells. However, dimerization and the subsequent nuclear translocation of IRF-3 were almost undetectable in CHC B cells. It has been demonstrated that TANK-binding kinase-1 (TBK1) and I*κ*B kinase-*ε* (IKK*ε*) are essential for the phosphorylation of IRF-3 [40]. The constitutive expression levels of both kinases were found to be markedly enhanced in CHC B cells. However, the reduced expression of TBK1 stabilizers, including Hsp90 [41] and DDX3X [42], and the enhanced expression of the IKK suppressor SIKE [43], were observed in CHC B cells, suggesting that IRF-3 phosphorylation was downregulated. Hence, transcription of the IFN-*β* gene was not augmented. These results strongly suggest that HCV infection circumvents innate antiviral immune responses, that is, type I IFN production in B cells, and (Figure 1) thus, takes advantage of B cells for persistent infection. 

 It can be assumed that, among B-cell subsets, memory B cells are the main reservoirs of HCV infection primarily because of their long lifespans. Supporting this notion, our recent study indicated that CD19^+^ CD27^+^ cells (memory B cells [44]) are recruited to the liver of patients with CHC through the interaction between CXCR3 expressed on CD19^+^ CD27^+^ cells and IP-10 (IFN-*γ*-inducing protein 10 kD) produced in the liver [45]. This strategy would be beneficial for HCV in securing sites for long-lasting infection. HCV infection of hepatocytes has long been considered an a* priori *assumption. However, this assumption does not necessarily mean that hepatocytes are the exclusive target of HCV infection. HCV may search for reservoir sites in other cellular compartments if the liver becomes unsuitable for replication, perhaps due to cellular destruction caused by the host immune response and/or by the development of conditions such as cirrhosis and hepatocellular carcinoma.

 Lymphoid reservoirs of HCV infection could play a role in viral persistence [29, 46–48]. Several maneuvers are employed for persistent infection of HCV [49]. Viral modulation is an effective strategy to escape host immune responses [50]. Another strategy is the suppression of the innate immunity of host by viral components. These components include HCV E2 protein, which acts as a decoy target of protein kinase R (PKR) [51]; HCV NS3/4A protein, which cleaves the adaptor molecules TRIF and IPS-1 and thereby blocks TLR3 and RIG-I signaling, respectively [52, 53]; HCV NS5A protein, which inhibits IFN-stimulated genes expression [54] and PKR function [55]; HCV core protein, which interferes with JAK/STAT signaling [56, 57]. Regardless of the mechanisms, the infection and replication of HCV in peripheral B cells should be considered barriers to the treatment of patients with CHC with antiviral regimens. Based on the notion that peripheral B cells serve as reservoirs for persistent HCV infection and from a therapeutic perspective, it may be beneficial to eliminate peripheral B cells in patients with CHC by the administration of anti-B-cell antibodies, such as rituximab, along with combination therapy with peginterferon and ribavirin to eliminate circulating HCV in the blood, leading to a synergistic effect on HCV clearance in patients with CHC.

## 4. HCV Infection and B-Cell Lymphomagenesis

 The striking association between HCV infection and type II mixed cryoglobulinemia (MC) has been well documented [4, 58, 59]. MC is a benign lymphoproliferative disorder and is regarded as a variant of low-grade B-NHL. Therefore, lymphotropism of HCV suggests that HCV could play a pathogenic role in the clonal proliferation of B cells [60, 61]. Because HCV RNA genomic sequences are not able to integrate into the host genome, indirect mechanisms of malignant transformation should be considered. In this regard, the persistent stimulation of B cells by viral antigens and/or the enhanced expression of lymphomagenesis-related genes could be responsible for leading to polyclonal and later to monoclonal expansion of B cells. Furthermore, the occurrence of a subsequent transformation may lead to B-NHL.

A number of epidemiological studies regarding the association between HCV infection and the occurrence of B-NHL have been carried out [5, 7, 62–65]. A substantial geographic as well as demographic variation exists in the association between HCV infection and risk of B-NHL. A positive association was found in Italy, Japan, and USA. A recent case-control study with a large number of subjects from the International Lymphoma Epidemiology Consortium based in Europe, North America, and Australia further confirmed the association between HCV infection and NHL and specific B-NHL subtypes, that is, diffuse large B-cell lymphoma (DLBCL), marginal zone lymphoma, and lymphoplasmacytic lymphoma [6]. In contrast, other studies from Northern Europe, UK, and Canada failed to show the association. Geographic differences in HCV genotype have been thought to cause these discrepancies [66] although this remains controversial. Large-scale, population-based, well-controlled studies are necessary to reach a robust conclusion. It can be concluded that, at least in areas with a high prevalence of HCV carriage, HCV is an important risk factor for B-cell lymphomagenesis.

 In this paper, we propose a novel hypothesis that peripheral B cells serve as reservoirs for persistent HCV infection. We also suggest that long-lasting HCV infection in B cells may induce lymphoproliferative disorders that may eventually evolve into B-cell NHL, although little is known about the mechanism responsible for B-cell lymphomagenesis. In the remainder of this section, the possible mechanisms of B-NHL tumorigenesis induced by HCV infection will be discussed based on current knowledge of lymphomagenesis-related genes.

 Activation-induced cytidine deaminase (AID) is essential for somatic hypermutation (SHM) and class switch recombination of immunoglobulin genes in B cells [67–69]. Recently, it has been proposed that AID may be instrumental in the initiation and progression of B-NHL. This is because a malfunction in either of the two processes stated above is apparently responsible for chromosomal translocations and aberrant SHM, which are the two main causes of genetic lesions associated with B-NHL [70, 71]. Several oncogenes have been demonstrated to be targets for SHM with immunoglobulin genes. In many cases, these anomalies activate the DNA damage response system that either allows DNA repair or eliminates the aberrant B-cell clones [72]. Failure of these repair systems may be a cause of B-cell malignancies. Specific features of SHM are the predominance of single-based substitution, the preference for transitions over transversion, and the specific targeting of the RGYW/WRCY motif. Pasqualucci et al. showed that hypermutation of proto-oncogenes exists in DLBCL [71]. However, in HCV-associated NHL, the number of mutations in some proto-oncogenes was lower than that already found in HCV-negative B-cell NHL patients [73]. Because there is a close association between HCV infection and the incidence of B-NHL, as described above [6, 7], analyzing the expression levels of AID in CHC B cells is of great interest.

 Lai et al. established a B-cell line (SB) from HCV-infected B-NHL cells. The virus particles produced from SB cell culture could infect primary human hepatocytes, Raji cells, and PBMC in vitro [74]. They examined the expression of AID in Raji cells and PBMC after HCV infection in vitro. It was found that HCV infection activated the expression of AID in Raji cells. AID expression level was also higher in PBMC of patients infected with HCV than in uninfected individuals [75]. However, their study did not assess which cell population showed an enhancement of AID expression. It was observed in our recent study that expression levels of AID mRNA were markedly enhanced in the CD19^+^, but not in the CD19^−^ subset of patients with CHC [32]. Furthermore, the enhanced expression of AID protein was detected in the CD19^+^ B-cell subset of patients with CHC [32]. The fact that this enhancement of AID expression is confined to the B-cell subset is extremely intriguing because several reports have demonstrated the augmented expression of AID in B-NHL [26, 76, 77]. 

 Using an AID-deficient mouse model, Pasqualucci et al. concluded that AID is required for germinal center-derived lymphomagenesis [78]. They addressed the issue of errors in AID-mediated antigen receptor gene modification processes being the principal contributors to the pathogenesis of human B-NHL. Increasing epidemiological evidence has highlighted the close correlation between HCV infection and B-NHL [6, 7, 79]. Thus, it is tempting to hypothesize that the enhancement of AID in CHC B cells is at least partly responsible for the initiation of lymphomagenesis. In fact, several recent studies suggest that AID is deeply involved in tumorigenesis [80–84]. Notably, HCV enhanced AID expression by NF-*κ*B activation through the expression of viral core proteins. Furthermore, NF-*κ*B expression was upregulated and activated by HCV NS2 proteins in HepG2 cells [85]. These findings suggest that inappropriate expression of AID acts as a DNA mutator that enhances genetic susceptibility to mutagenesis [86]. 

 Additionally, enhanced expression of other lymphomagenesis-related genes including cyclin D1, cyclin D2, BAL, STK15, and galectin-3 in CHC B cells is worth considering [32]. Overexpression of CCND1, which alters cell-cycle progression, is frequently observed in various tumors and may contribute to tumorigenesis [87, 88], whereas CCND2 is known to be expressed at constitutively high levels in B-NHL [89]. BAL is a novel risk-related gene in DLBCL, a typical B-NHL [90], while STK15 is a gene highly expressed in a histologically aggressive type of NHL [91]. Galectin-3 is an antiapoptotic protein, highly expressed in DLBCL [92]. Presumably, the enhanced expression of these genes in CHC B cells [32] may also correlate with B-cell lymphomagenesis.

 Tumor necrosis factor alpha-induced protein 3, also called A20, was first identified in 1990 as a TNF-induced cytoplasmic protein with zinc finger motifs [93], which thereafter has been described as a key player in the negative regulation of inflammation by terminating NF-*κ*B signaling [94–96]. Recently, A20 has gained much attention as a novel tumor suppressor [97, 98]. Honma et al. first reported that A20 is frequently inactivated or even deleted in mantle cell lymphoma and DLBCL, and they raised the possibility that inactivation of A20 may be at least partly responsible for lymphomagenesis [99, 100]. Other investigators have subsequently supported their findings [101, 102]. Moreover, A20 also regulates antiviral signaling as well as programmed cell death [103–105]. Currently, the expression, biological activities, and mechanisms of action of A20 have been the focus of attention on a wide scale [106]. Interestingly, Ngueyn et al. reported [107] that the HCV core protein induced an increased expression of A20 in the human hepatocyte cell line HepG2, which has generated a genuine interest in the expression of A20 in peripheral B cells of patients with CHC. Our preliminary data suggests that the A20 molecule is partially cleaved in CHC B cells (Kusunoki et al.; in preparation). An intriguing possibility is that the A20 gene interacts with and is mutated by AID, the expression of which is dramatically enhanced in CHC B cells [32]. In this regard, the expression levels of A20 in patients with B-NHL suffering from chronic HCV infection are worth investigating.

## 5. Conclusion

**Figure 2:**
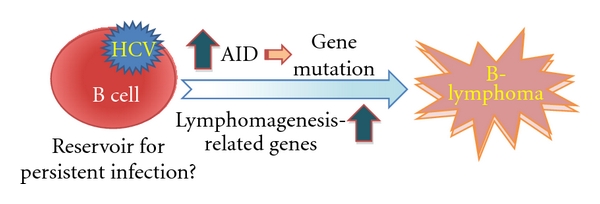
Role of HCV infection in B-cell lymphomagenesis, a hypothesis.

In this paper, we summarized recent studies illuminating the possible role of HCV infection in B-cell lymphomagenesis. We proposed a hypothesis that HCV utilizes B cells as reservoirs for persistent infection, which could result in the enhanced expression of lymphomagenesis-related genes, particularly AID, which is thought to be crucial for the initiation and progression of B-NHL (Figure 2). Elimination of HCV in plasma by antiviral reagents as well as in peripheral B cells by specific antibodies would be beneficial for patients with CHC to achieve a complete viral clearance. Finally, although a positive association between HCV infection and B-NHL occurrence is still being debated [108–111], it is worthwhile to investigate the possible mechanisms by which B-cell lymphoproliferative disorders, which may evolve into B-NHL, are induced in patients with CHC.
